# Evaluation of a gene information summarization system by users during the analysis process of microarray datasets

**DOI:** 10.1186/1471-2105-10-S2-S5

**Published:** 2009-02-05

**Authors:** Jianji Yang, Aaron Cohen, William Hersh

**Affiliations:** 1 Department of Medical Informatics and Clinical Epidemiology, Oregon Health & Science University, Portland, Oregon 97239, USA; 2 Portland Center for the Evaluation of Clinical Services, Portland VA Medical Center, Portland, Oregon 97239, USA

## Abstract

**Background:**

Summarization of gene information in the literature has the potential to help genomics researchers translate basic research into clinical benefits. Gene expression microarrays have been used to study biomarkers for disease and discover novel types of therapeutics and the task of finding information in journal articles on sets of genes is common for translational researchers working with microarray data. However, manually searching and scanning the literature references returned from PubMed is a time-consuming task for scientists. We built and evaluated an automatic summarizer of information on genes studied in microarray experiments. The Gene Information Clustering and Summarization System (GICSS) is a system that integrates two related steps of the microarray data analysis process: functional gene clustering and gene information gathering. The system evaluation was conducted during the process of genomic researchers analyzing their own experimental microarray datasets.

**Results:**

The clusters generated by GICSS were validated by scientists during their microarray analysis process. In addition, presenting sentences in the abstract provided significantly more important information to the users than just showing the title in the default PubMed format.

**Conclusion:**

The evaluation results suggest that GICSS can be useful for researchers in genomic area. In addition, the hybrid evaluation method, partway between intrinsic and extrinsic system evaluation, may enable researchers to gauge the true usefulness of the tool for the scientists in their natural analysis workflow and also elicit suggestions for future enhancements.

**Availability:**

GICSS can be accessed online at:

## Background

Gene microarray technology is frequently used in biomedical research to investigate the differential expression levels of genes in the whole genome under different conditions, e.g. control vs. diseased, young vs. aged. For instance, experiments can be performed to conduct a comparison of gene expression between normal and breast cancer tissues. These results are already translating into changes in clinical practice [1]. Since these experiments can measure the expression level of tens and thousands of genes simultaneously, the analysis of the results produced is nontrivial because of the large data size.

Searching the literature databases such as MEDLINE for information on the genes differentially expressed is a necessary task for translational researchers during the analysis of the microarray experiment. With the increasing volume of published full-text scientific articles, even the most robust information retrieval (IR) engine returns more documents than scientists are able to manually review. One approach to address this issue is to automatically produce customized summaries for the users who are analyzing the result of a specific microarray experiment. Summarization is defined by Sparck Jones [2] as "*a reductive transformation of source text to summary text through content reduction selection and/or generalization on what is important in the source"*. Automatic summarization systems have been studied since the late 1950s [3,4] and applied in different domains with some notable success [5], but less well studied in the biomedicine domain [6]. The information that is of most interest to scientists may reside in sentences describing some specific biological process such as *phosphorylation *and *activation*, or the relationship between genes and a certain medical conditions. These specific information requirements can be used in the biomedical domain by emphasizing domain-specific keywords to extract important information and to construct summaries.

By exploiting the use of domain terminology and the analysis workflow of microarray experiments, we adapted the automatic summarization technology of Edmundson [3] to the biomedical domain. Focusing on the analysis of differentially expressed gene sets from microarray data, the Gene Information Clustering and Summarization System (GICSS) consists of a two-step process. First the gene set is clustered into functional related groups based on free text, Medical Subject Headings (MeSH), and Gene Ontology (GO) terms. Next, a summary for each gene is generated as sentences ranked by features such as domain vocabulary, length, representation of its functional cluster, cue words and recency. This is a novel approach, since previous work either focus on functional gene clustering [7,8] or gene information summarization[9], but there was no integration of these two related steps in microarray data analysis process.

Evaluation is a critical part of any system development. Since the ultimate goal of a summarizer is to present the succinct information in the literature to practicing biomedical researchers, *extrinsic evaluation *that measures how useful the system is to the intended end users has been heralded by experts in the field [10]. However, text-mining and automatic summarization systems are still lagging behind information retrieval systems in routine use, and user-oriented evaluation presents greater challenges in labor intensiveness and study design than *intrinsic evaluation *(measures how good the system is) [10]. In the evaluation of this project, biomedical researchers served as the subjects in evaluating the output of our systems in a real-world context using their own microarray data. This allowed us to create a gold standard and use intrinsic evaluation measurements. The experts judged how well the clustering and sentence ranking algorithms work with the microarray experiment data they were analyzing at the time of evaluation. Our approach is a hybrid paradigm, providing measures based on actual tasks important to the subjects with reduced human labor. This is one-step closer to the ultimate goal of real-time, user-oriented evaluation.

## Methods

### System overview

**Figure 1:**
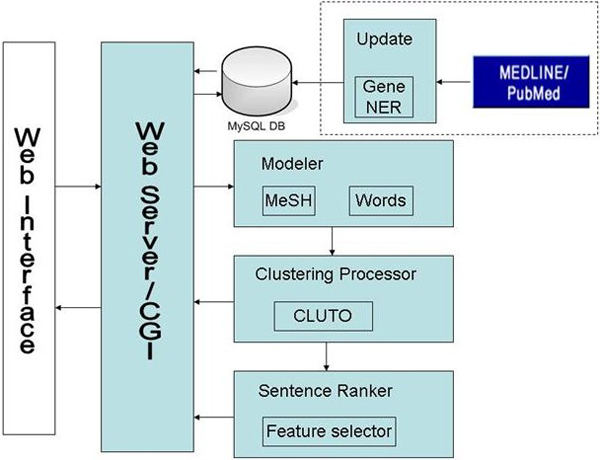
**System architecture**. The system's core components consist of a GO, MeSH and word pre-processor, a wrapper around CLUTO1 (a preexisting clustering application), and a sentence ranker.

The details of the system implementation can be found in [11]. In brief, GICSS consists of a GO, MeSH and word pre-processor, a wrapper around a clustering application CLUTO [12], and a sentence ranker (Figure 1). The system has two gene and protein name entity recognition and normalization systems (NER) [13] that can be interchanged to focus on either mouse genes or human genes. We decided to focus on mouse genes initially to allow us to restrict the domain to mouse research, an area that we had developed relationships with researchers who worked on mouse disease models. From this point on, the system is described in terms of mice gene summarization branch, while the human gene branch shares identical modules except for the gene NER.

### Clustering of genes into functionally related groups

We modeled the genes by three categories of features: MeSH Headings, GO terms and text words in the sentences with at least one reference to a gene. Each gene is modeled as vector combination of the above three categories of features. Direct k-means clustering algorithm was used to find the functional clusters.

### Ranking of sentences for each gene

Sentences are modeled as word vectors after parsing, stop word removal and stemming. Each sentence is assigned a score by linear combination of features. Specifically, sentence score *S *is calculated as:

*S *= *w*_1_*CluSim *+ *w*_2_*NGene *+ *w*_3_*CTword *+ *w*_4_*TPword *+ *w*_5_*L *+ *w*_6_*Recency*

• Cluster representation (*CluSim*). The top five descriptive features (a set of MeSH, GO terms and/or text words) for each gene cluster from the previous step are used as this ranking measure.

• Gene relations (*NGene*). Sentences referenced to more than one gene/protein names score higher, otherwise, 0.

• Cue phrases (*CTword*). This is identical to the Edmundson's Cue feature based on the assumption that the importance of a sentence is represented on the presence of certain key terms. For example, the term 'conclusion' may indicate importance.

• Domain specific keywords (*TPword*). Biologically relevant keywords were extracted from the Textpresso [14] ontology.

• Length (*L*). *L *is calculated as the fraction of the longest sentence.

• *Recency *is calculated as a linear scale for the sentences from one to zero.

The weighting scheme of *w *was adjusted empirically based on feedback from users during the first stage of the evaluation process. This used a data set distinct from those used for the evaluation discussed below.

### Evaluating the clustering algorithm

**Figure 2:**
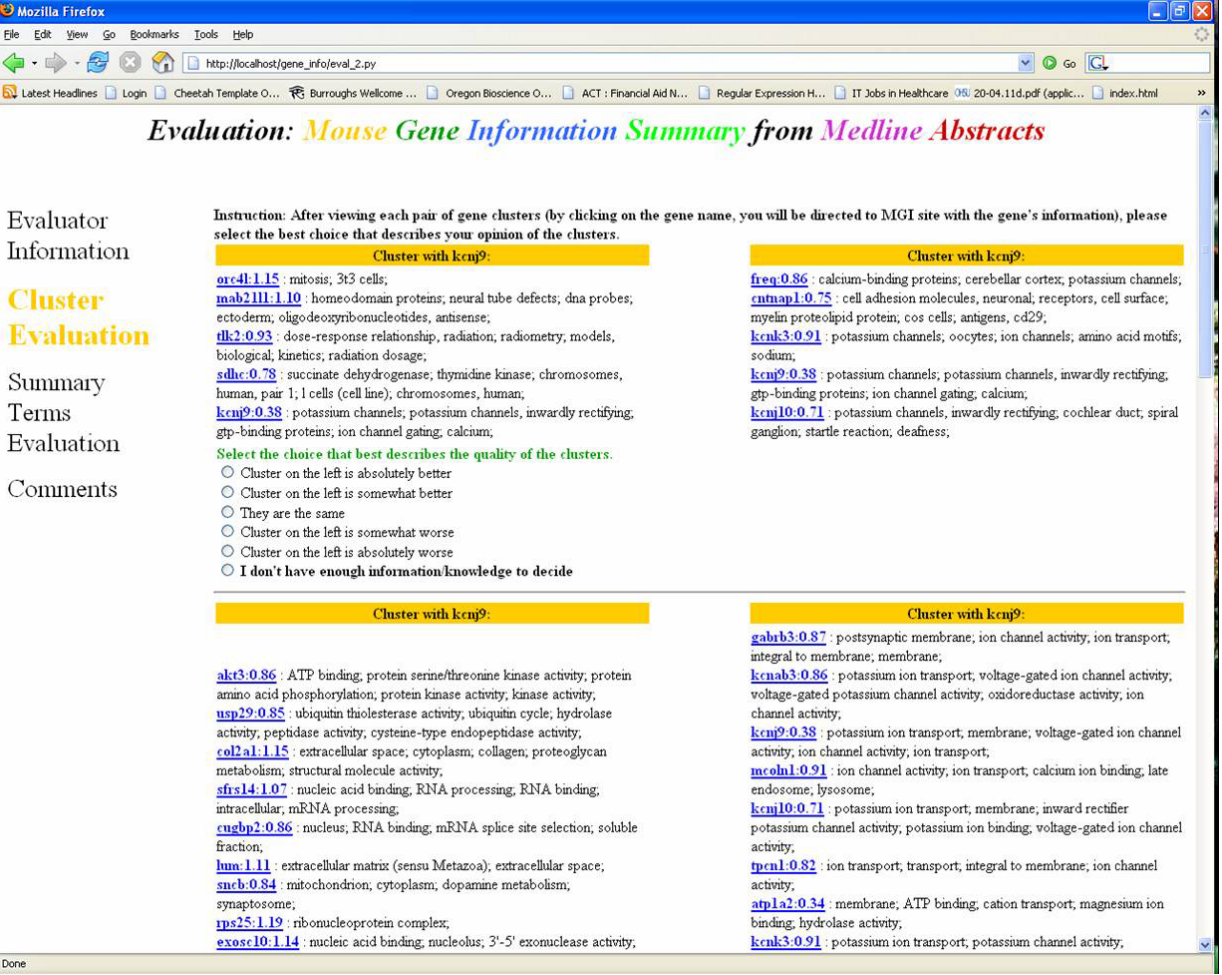
**Screen shot of the evaluation web page**. Evaluation was done with web survey forms, while the users were analysing the microarray experiment data with GICSS system output.

Five gene sets from the results of five different microarray experiments were tested on the GICSS system by OHSU-based mouse genomic researchers. Each person rated the gene set generated by his or her own lab. For each gene set, the participants labeled the genes they were familiar with. Each of the participants compared cluster pairs, which contained at least one of the familiar labeled genes. This setup had the scientists work on the genes they picked to ensure that they had the expertise for the particular gene cluster in order to adequately judge the result. First, participants judged the validity of two clusters for each gene set by comparing clusters with random groupings also including the familiar gene. Then, the top five summary/descriptive terms for each cluster were shown to the participants and they were asked to change their judgment if needed after seeing the summary terms. For each cluster pair, participants chose the more useful cluster of genes from the pair using a 5-point Likert scale, with the center point being no preference. The participants can also indicate that he/she was not able to decide the quality of the cluster pair. The left/right order of the clusters was randomized at run-time during the evaluation. A screen shot of the evaluation web page is presented in Figure 2. The results for cluster usefulness were analyzed using two-way ANOVA to assess the effects of both the participants and the clustering features. The results for comparison of rankings before and after showing the cluster keywords were analyzed by Wilcoxon signed rank test.

### Evaluating ranking of informative sentences

Sentences for ten genes (genes from each of the clusters evaluated in the previous step) were used in this step. Sentences from the output of the system and PubMed searches were pooled together and judged by the same scientists who studied the gene sets. The searches on PubMed were done by e-search provided by Entrez [15]. The queries were the name of the gene and synonym expansion using the synonym dictionary from [13]. The results of the searches were filtered on Date of Publication (DP) to limit to the time period of 1994–2003 and on MeSH term (MH) 'Mice'. These filtering criteria were the same as the text collection, making the comparison between PubMed search results and system output possible.

The raters assigned an R (relevant) or NR (not relevant) label to each pooled sentence by judging if it had relevant information for understanding the specific gene studied in the microarray experiment they were analyzing. Results from the first two genes were used to hand-tune the ranking parameters and the other eight were analyzed to study the system. Three sentence presentations were compared by average precision (AveP) using the relevance judgments as a gold standard:

1. Our system output: Sentences with reference to the gene extracted from the abstracts ranked by the scoring algorithm.

2. Same sentence set as in #1 above but in reversed chronological order (same as PubMed's ranking).

3. Output from PubMed search (title of abstract in reversed chronological order).

For each sentence presentation, the AveP scores were calculated for each of the genes and the scores were analyzed using repeated measure with post-hoc comparison with the Sidak adjustment.

## Results

### Gene clustering

The five gene sets evaluated were results from microarray experiments covering different areas of translational research such as mice model of myelodysplastic syndromes and genes in the brain circuits involved in drug dependence and withdrawal. They were obtained from similar platforms: Affymetrix 430A or 430 2.0. The number of clusters for each gene set depended on the number of genes in the list and the natural diversity of the gene set. The size of the five gene sets ranged from 53 to 275, which we believed represented the numbers of differentially expressed genes produced in microarray experiments. However, the criteria to choose these sets of differentially expressed genes were chosen individually by each scientist for their own data, without any intervention from the author. The number of genes in the 10 clusters evaluated by the scientists ranged from four to 12.

**Figure 3:**
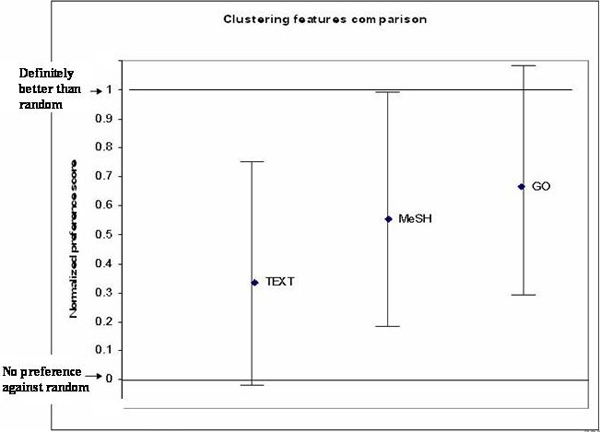
**Effect of features in building gene clusters**. The comparison of the three features (Text, MeSH and GO) for their usefulness for building gene clusters for the analysis of microarray dataset. Normalized preference at zero indicates no preference versus random grouping, while one indicates absolutely better than random.

Two-way ANOVA model analysis of cluster usefulness indicated no significant difference among the three features (MeSH, GO and text) and judges. In Figure 3, marginal means for each feature showed that both GO and MeSH were significantly better than zero (here, zero is equivalent to random grouping).

**Figure 4:**
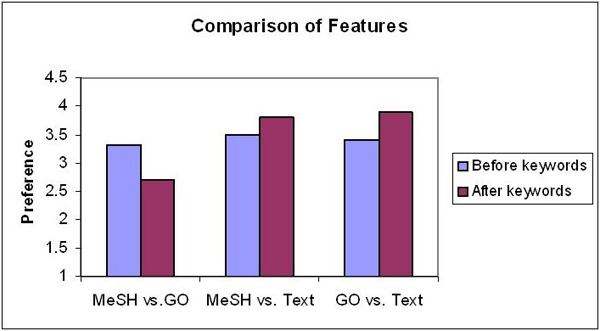
**Effect of keywords for cluster preference**. Side-by-side comparison of cluster preference, before and after showing the keywords to the participants.

Wilcoxon's signed ranks test on the before-and-after-keyword paired tests indicated that presentation of keywords did not significantly influence the preference of cluster choices for all of the three features (Figure 4). While the difference was not significant, the preference scores for GO clusters and MeSH clusters increased over text clusters after showing the keywords.

### Sentence ranking

AveP scores for PubMed search results were consistently much lower than GICSS output. GICSS output was also consistently better than ranked by recency sentence presentation with only one exception in gene *kcnj9 *(Table 1). The ranking algorithm in GICSS (as judged by mean average precision-MAP) gave a 3.3% increase over reverse chronological order of sentences and close to ten-fold increase above PubMed titles. Repeated measure analysis of AveP suggested that overall there was significant difference among the three sentence presentations (p < 0.001). Post-hoc analysis with Sidak adjustment for multiply comparisons indicated that the GICSS system output was significantly better than PubMed (p < 0.001). However, the difference between the GICSS system output and sentence presentation ranked by recency was not significant at the 0.05 level (p = 0.08).

**Table 1:** Average precision scores for the three sentence presentations. The GICSS system performed significantly better than PubMed titles presentation.

**Gene**	**GICSS**	**Recency**	**PubMed**
adamts1	0.580264	0.534627	0.039064
usp18	0.921985	0.900525	0.103968
irak3	0.654296	0.615089	0.033333
cxcl12	0.854571	0.843545	0.000000
kcnj9	0.647421	0.666607	0.058620
pglyrp1	0.780637	0.717311	0.117634
ptx3	0.794350	0.783601	0.047008
clca1	0.683284	0.667058	0.205523
**MAP**	**0.739601**	**0.716046**	**0.075644**

**Figure 5:**
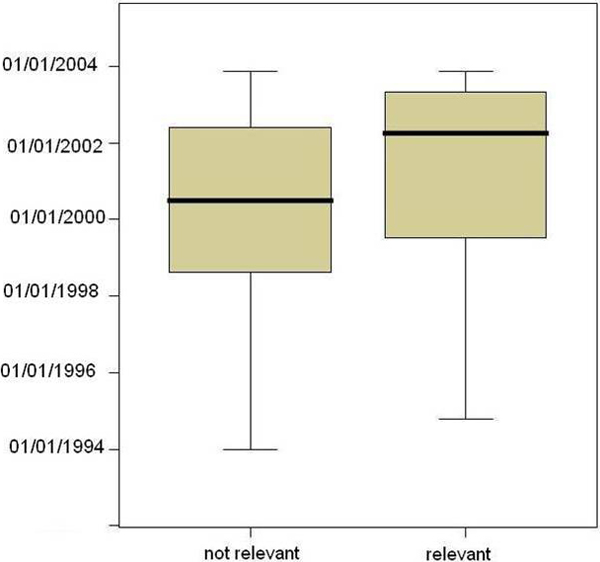
**Relation between recency and relevancy**. Box plot graph of the distributions of relevant and not-relevant sentences over date of publication (DP). The median for DP for the relevant sentences is in April 2002, while DP for non-relevant sentences is July 2000.

The relationship between recency and the probability of being judged as a relevant sentence was further investigated. Judged sentences for all ten genes were pooled and separated into either relevant or not-relevant groups. There were a total of 578 sentences with 357 judged relevant and 221 judged not relevant. The distribution of both groups over date of publication (DP) was studied. The box plot of the two distributions indicated that the median difference between date of publication (DP) for relevant and not-relevant sentences is close to two years. This indicated that the more recent the sentence, the more likely it is judged as relevant by the participants in this evaluation study (Figure 5).

## Discussion

### Comments on the system

In general, the clustering algorithm gave better gene groups than random as supported by the fact that clusters generated by both MeSH and GO terms were significantly better than random grouping of genes. The comparison between the feature types showed insignificant differences; even though the confidence interval trend suggested that MeSH and GO may be better than text as features for clustering. It appeared that the perception of a good cluster did not depend on the scientist knowing the clusters' descriptive terms. Once the participants found the better cluster, they were likely to stick with it even after seeing the keywords. The preference for MeSH and GO clusters suggests that controlled vocabularies fare better than text words for generating clusters. Controlled vocabularies usually have more domain-specific content, which may be able to give more information to users.

Providing sentences in the abstract gave much more relevant information than titles alone. The results showed significant differences between the system output and the PubMed search output. These results suggest that it may be useful for the PubMed result list to include highlighted keywords from the sentences in the abstracts to provide more information to the searchers. In the current title-only list, some relevant articles may be missed because the titles do not provide enough information to warrant further exploration of the abstract, especially when the returned list is long.

Further analysis of the relation between recency and relevance indicated that the more recent the date of publication, the more likely the sentence be judged as relevant. This relation may be explained by the fact that the experts doing the relevance judgment were to a certain extent aware of the knowledge accumulation timeline of the specific genes. It seems that this is a good assumption because the experts were instructed to select two genes they were familiar with to perform the evaluation. Because of their pre-existing knowledge of the genes, they were likely to pick the newly discovered information as more relevant than the well-known facts on the genes.

GICSS is built in a modular fashion. This open framework allows the substituting of other NER systems for changing the method and species. Furthermore, the sentence ranking approach can be enhanced with new useful features as they become apparent.

### Reflection on the evaluation process

The time for cluster evaluation of each gene set ranged from 20 to 35 minutes. During the experiment, we found that judging cluster pairs was not an easy task for the scientists. Even though each cluster had at least one of the genes they chose as familiar, it was very common that some genes in the cluster (with average size of 8 genes) were not very familiar to them. In order to judge the quality of the cluster, they needed to follow links in the evaluation screen to obtain information on other genes in the cluster. This created a larger than expected work load for the evaluators and by the end of the session, they make their best judgments without going through the information for not-so-familiar genes, possibly due to fatigue. On the other hand, without a system such as GICSS which provided hyperlinked text to articles relevant to genes and clusters, the scientists would have a much larger workload for making any sense of the clusters at all. Systems that only provide gene cluster output effectively leave scientists in this situation.

The workload was also the reason each participant was asked to judge clusters for only two genes, which amounted to judging 12 cluster pairs in our experimental design. The fatigue factor may also have influenced the quality of the judgment. In order to overcome the difficulty of getting experiment participants and ensure the quality of the judgment, it will be better to conduct future evaluation experiments in a real-world routine user setting, spread over a longer period of time where the scientists are analyzing their data in the normal course of their gene microarray investigations. For example, the system could log the clicking and timing of the participants in addition to the defined questions presented to the participants. In this manner, we may be able to capture some of the information that might have been lost during this evaluation. For example, evaluators mentioned that after reading the summary sentences they have found informative abstracts that were important to their analyses. These examples were important to illustrate the system's success, but they were not recorded for evaluative purposes because the relevance judgments were the focus of the sentence extraction evaluation.

How to best measure the quality of clusters is still a general issue, especially in this case, where we define quality as how useful the clusters were for the analysis of a specific microarray experiment. Some analytical measures, such as internal and external similarities, entropy and mutual information, are likely not correlate closely. These measures are commonly used to quantify the quality of the clusters in many comparative experiments [7,16]. We are unaware of any previous work on how the purity of the cluster as defined by these statistical measures correlates with the biological meaning of the clusters for a user. Furthermore, in this study, the raw differentially expressed gene list from the experiment was clustered. By nature, the gene list contains genes that were differentially expressed and most likely from many different pathways and groups especially considering many genes may perform multiple functions. We expect the list to be harder to cluster than choosing several distinct known gene groups, such as GO and cell cycle groups and try to cluster them to the right class, which is the most common evaluation method used [7,16,17].

The GICSS system can support the use of user defined query terms in selecting the important sentences. In this way, it gives the users more leverage in getting to the information of greatest interest. For example, they can enter a disease's name in order to retrieve sentences referring to the gene and the disease. Hopefully, this feature can provide customized sentences presentation to fit different needs of the users. Due to the limited resources in this project, this feature was not evaluated and the studying of its use is future work.

## Conclusion

We built and evaluated a gene information summarization system for translational researchers. The evaluation was performed during the time that real scientists were analyzing their own microarray experiment results. It was a hybrid evaluation method, partway between intrinsic and extrinsic system evaluation, and may enable researchers to more truly gauge the usefulness of a system to its intended users. The result of the evaluation suggested the system could be a useful tool for translational genomics researchers.

## Competing interests

The authors declare that they have no competing interests.

## Authors' contributions

JY conceived and planned the project, developed GICSS, designed and conducted the evaluation, performed data collection and analysis, and authored the manuscript. AC and WH provided advice in project planning, evaluation method design and manuscript editing.
